# Colorimetric determination of trace orthophosphate in water by using C_18_-functionalized silica coated magnetite

**DOI:** 10.1038/s41598-021-02516-4

**Published:** 2021-11-29

**Authors:** Vanpaseuth Phouthavong, Supone Manakasettharn, Duangkamon Viboonratanasri, Siriwit Buajarern, Panida Prompinit, Kamonthip Sereenonchai

**Affiliations:** 1 Department of Chemistry, Faculty of Science and Technology, Thammasat University, Pathumthani, 12120 Thailand; 2 Department of Chemistry, Faculty of Natural Sciences, National University of Laos, P. O. Box 7322, Vientiane, Lao PDR; 3 National Nanotechnology Center (NANOTEC), National Science and Technology Development Agency (NSTDA), Thailand Science Park, Pathumthani, 12120 Thailand; 4 Flow Innovation-Research for Science and Technology Laboratories (FIRST Labs), Bangkok, Thailand

**Keywords:** Analytical chemistry, Environmental chemistry, Green chemistry

## Abstract

In this study, we customized magnetic sorbents by functionalizing silica coated magnetite with octadecyl(C_18_)silane (Fe_3_O_4_@SiO_2_@C_18_). This sorbent was intended for the determination of trace orthophosphate (*o*-PO_4_^3−^) in unpolluted freshwater samples. The *o*-PO_4_^3−^ was transformed to phosphomolybdenum blue (PMB), a known polyoxometalate ion. Then the PMB were coupled with cetyl trimethyl ammonium bromide (CTAB), cationic surfactant, in order to hydrophobically bound with the Fe_3_O_4_@SiO_2_@C_18_ particles through dispersive magnetic solid-phase extraction (d-MSPE) as part of sample preconcentration. The PMB–CTAB–magnetic particles are simply separated from the aqueous solution by the external magnet. The acidified ethanol 0.5 mL was used as PMB-CTAB eluent to produce an intense blue solution, which the absorbance was measured using a UV–Vis spectrophotometer at 800 nm. The proposed method (employing 2 mg of Fe_3_O_4_@SiO_2_@C_18_) yielded an enhancement factor of 32 with a linear range of 1.0–30.0 µg P L^−1^. Precision at 6.0 µg P L^−1^ and 25.0 µg P L^−1^ were 3.70 and 2.49% (RSD, n = 6) respectively. The lower detection limit of 0.3 µg P L^−1^ and quantification limit of 1.0 µg P L^−1^ allowed trace levels analysis of *o*-PO_4_^3−^ in samples. The reliability and accuracy of the proposed method were confirmed by using a certified reference material. Our method offers highly sensitive detection of *o*-PO_4_^3−^ with simple procedures that can be operated at room temperature and short analysis time.

## Introduction

Phosphorus is a growth-limiting nutrient in aquatic systems. It is present at trace levels of between 10–50 µg P L^−1^ in most natural clean water^1^. If there are excessive amounts of phosphorus, overgrowth of algae rapidly occurs, creating an environmental problem. Orthophosphate (*o*-PO_4_^3−^) is the predominant species in most water resources and is also the most bioavailable form of phosphorus^2^. It is therefore essential to develop a quick, sensitive and reliable methods for monitoring trace *o*-PO_4_^3−^ so we can prevent the problem from possible causes readily.

Quantification of *o*-PO_4_^3−^ with the molybdenum blue method normally implement the preconcentration procedures in order to improve sensitivity. Various preconcentration techniques have been developed such as cloud-point extraction (CPE)^3,4^, suspended droplet micro-extraction (SDME)^5^, dispersive liquid–liquid micro-extraction with solidified floating organic drop micro-extraction (DLLME-SFODME)^6^, electrostatically induced stoichiometric extraction (EISE) via layered-double hydroxides (LDHs)^7^, vortex-assisted natural deep eutectic solvent micro-extraction (VA-NADES-ME)^8^ and, more recently, vortex-assisted based supramolecular solvents-dispersive liquid–liquid micro-extraction (VA-SS-DLLME)^9^. Even though the techniques offer low limits of detection and high enhancement factor however, the procedures are not simple and handling the liquid extractant require certain skills. Another approach that not only improve the sensitivity but also simplifies the procedures is to exploit solid-phase extraction (SPE) with different types of solid sorbent^10–15^. Those solid sorbent particles can be classified into two groups: non-magnetic and magnetic.

Non-magnetic particles with C_18_–functionalized surfaces have been employed in both off-line and on-line extraction for *o*-PO_4_^3−^ determination in aqueous samples^10,15^. First the *o*-PO_4_^3−^ was converted to phosphomolybdenum blue complex (PMB) and followed by formation of a neutral ion pair complex with cationic surfactants such as cetyltrimethylammonium bromide (CTAB)^10^ and dodecyltrimethylammonium bromide (DTAB)^15^. Then the neutral ion pair complex (PMB-CTAB or PMB-DTAB) was adsorbed onto the C_18_ particles via hydrophobic interaction before the adsorbed C_18_ particles were collected. Finally, elution of PMB from the collected C_18_ particles was analyzed through colorimetric detection.

Special interest has been given to the application of magnetic materials in so-called magnetic solid-phase extraction (MSPE). Commonly the small particle size of particles possesses large surface-to-volume ratio which allows high recovery of analyte. The main advantage of MSPE is that the magnetic particles can be separated from the large volume of solution efficiently and effortlessly by external magnet^16^. One of the widely used magnetic particles is bare magnetite (Fe_3_O_4_), this iron oxide has stronge magnetism, low toxicity, and very easy to synthesize^16–18^. However, there are some drawbacks^16,17^ such as ease of aggregation, deterioration of the magnetic properties when exposed to air, dissolution in strongly acidic solutions and the major drawback of magnetite is lack of sorption specificity. Thus, application of bare magnetite in limited especially when the application requires strongly acidic condition (pH < 1). So, modification of Fe_3_O_4_ by coating with protective layers while maintaining the magnetic properties and large surface-to-volume ratio can overcome this constraint. Octadecyl (C_18_) silane modified magnetite (Fe_3_O_4_@SiO_2_@C_18_) is one of the most widely used lipophilic adsorbent^19,20^ for extraction/preconcentration of hydrophobic/nonpolar compounds^19–24^ since it provides strong hydrophobic affinity and excellent stability^19,25^. However, it has not been employed for *o*-PO_4_^3−^ determination.

Determination of *o*-PO_4_^3−^ were diverged to analysis of *o*-PO_4_^3−^ directly and transform *o-*PO_4_^3−^ into polyoxometalate species (POMs) which offer many novel analysis strategies because the chemical nature of POMs is pH dependent^26^. Since POMs are negatively charged, our strategy is to couple PMB with cationic surfactant, leading to electrostatic interactions between both chemicals and then utilizing hydrophobic interaction between surfactant and Fe_3_O_4_@SiO_2_@C_18_ to effectively extracted PMB from the sample solutions.

**Figure 1:**
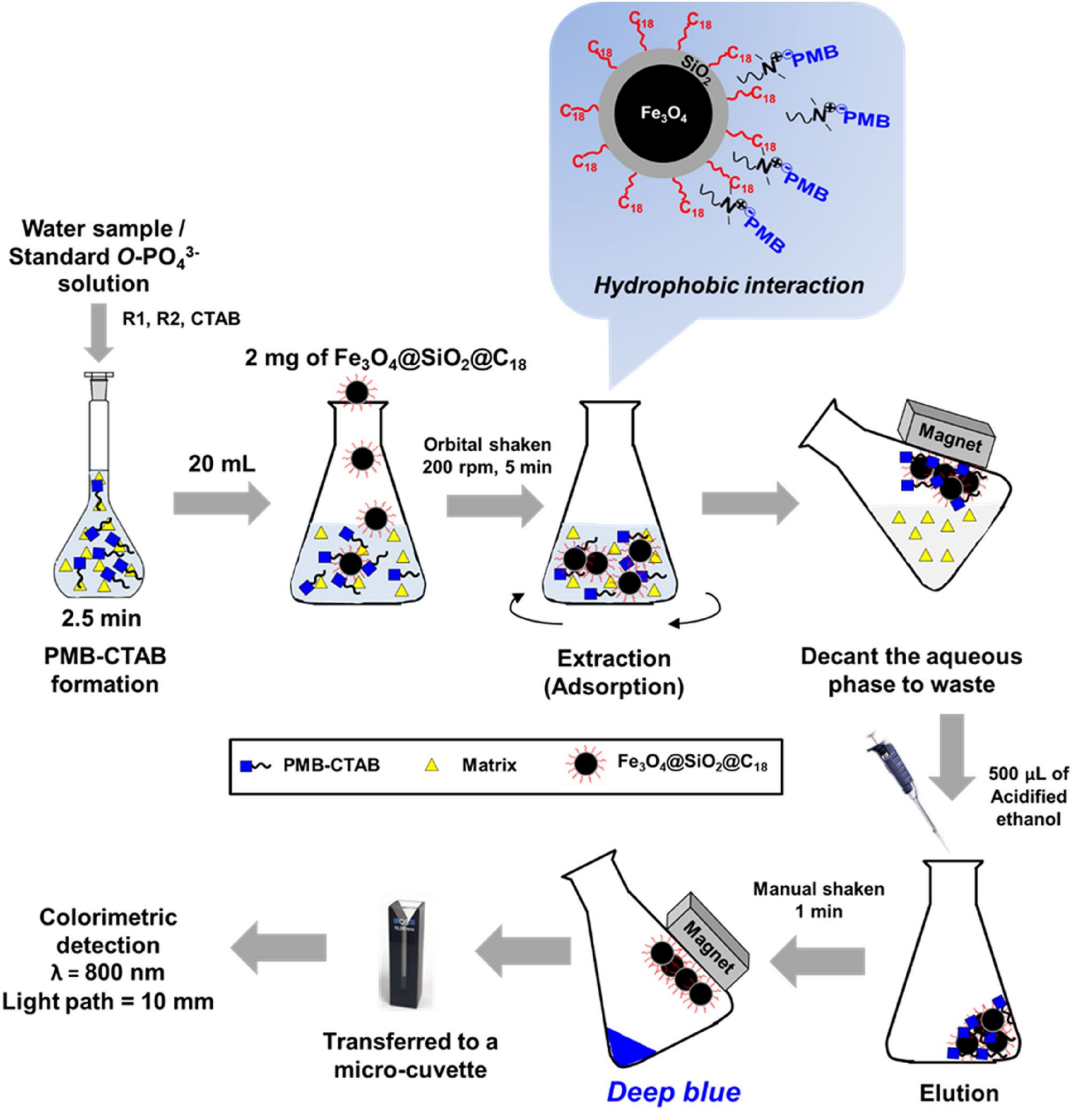
Schematic illustration of d-MSPE procedure for determination of *o-*PO_4_^3−^ as PMB–CTAB complex using Fe_3_O_4_@SiO_2_@C_18_.

In this study, we developed a method based on dispersive magnetic solid-phase extraction (d-MSPE) for simple, fast, specificity and highly sensitive detection of trace *o*-PO_4_^3−^ focusing in freshwater. The Fe_3_O_4_ magnetic particles were coated by silica (SiO_2_) layer to produce Fe_3_O_4_@SiO_2_ particles which still possess magnetic property and can tolerate strongly acidic condition (pH < 1). To increase selectivity and affinity of the Fe_3_O_4_@SiO_2_ particles to the neutral ion pair complex, PMB–CTAB, the silica layer was functionalized with octadecyl (C_18_) silane, producing Fe_3_O_4_@SiO_2_@C_18_ particles (see Fig. 1). The structural properties of the synthesized Fe_3_O_4_@SiO_2_@C_18_ particles, including their adsorption capacity towards PMB–CTAB, were studied and then used for developing a protocol for *o*-PO_4_^3−^ determination in freshwater samples. The reusability and storage durability of the synthesized Fe_3_O_4_@SiO_2_@C_18_ particles for *o*-PO_4_^3−^ analysis was also investigated. Finally, the proposed method was validated and applied to trace *o*-PO_4_^3−^ detection in river, canal, and tap water samples.

## Experimental

### Chemicals

All chemicals were of analytical grade and used without further purification. Reagents were prepared with Type I deionized water (18 MΩ·cm^−1^, ELGASTAT UHQ PS., ELGA, England).

For preparation of Fe_3_O_4_@SiO_2_@C_18_ particles, the following chemicals were used: iron (II) chloride tetrahydrate (FeCl_2_∙4H_2_O) (Sigma-Aldrich, Germany), iron (III) chloride hexahydrate (FeCl_3_∙6H_2_O) (Sigma-Aldrich, Germany), 30% (w/v) ammonia solution (Carlo Erba, Italy), absolute ethanol (Carlo Erba, Italy), tetraethyl orthosilicate (TEOS) (Fluka, Switzerland), anhydrous pyridine (Sigma-Aldrich, Germany), and chloro(dimethyl)octadecylsilane (Sigma-Aldrich, USA).

A 100 mg P L^−1^ stock solution was prepared by dissolving 0.5624 g of potassium dihydrogen phosphate (KH_2_PO_4_) (Merck, Germany) in deionized water. Mixed reagents (R_1_) were prepared in a 50-mL volumetric flask, by sequentially dissolving 5 mL of sulfuric acid (98% (w/w), H_2_SO_4_) (RCI LabScan, Thailand), 0.0116 g of potassium antimony tartrate hemihydrate (C_4_H_4_KO_7_Sb∙1/2H_2_O) (Carlo Erba, Italy), and 0.4325 g of ammonium molybdate tetrahydrate ((NH_4_)_6_Mo_7_O_24_∙4H_2_O) (Carlo Erba, Italy) in deionized water. A 100 g L^−1^ ascorbic acid reducing agent (R_2_) was prepared by dissolving 5.00 g ascorbic acid (Carlo Erba, Italy) with deionized water in a 50-mL volumetric flask. The CTAB solution (0.25 g L^−1^) was prepared by dissolving 0.1250 g of CTAB (Ajax Finechem, New Zealand) in 500 mL deionized water. Acidified ethanol was prepared by dissolving 30.4 mL of 98% (w/w) H_2_SO_4_ in absolute ethanol to a final concentration of 0.56 mol L^−1^ ^10^.

The following chemicals were used for the interference study: sodium metasilicate anhydrous (Na_2_SiO_3_) (Fluka, Switzerland), sodium arsenate dibasic heptahydrate (HAsNa_2_O_4_∙7H_2_O) (Sigma Aldrich, India), sodium chloride (NaCl) (Carlo Erba, Italy), sodium hydrogen carbonate (NaHCO_3_) (Carlo Erba, Italy), sodium nitrite (NaNO_2_) (Ajax Finechem, New Zealand), potassium nitrate (KNO_3_) (Carlo Erba, Italy), magnesium sulfate anhydrous (MgSO_4_) (Panreac, Spain), and potassium dichromate (K_2_Cr_2_O_7_) (Carlo Erba, Italy).

A certified reference material (CRM) for *o*-PO_4_^3−^ (product ID QC1166; certified value 0.752 ± 0.0140 mg P L^−1^; acceptance interval 0.526–0.978 mg P L^−1^; traceable to NIST SRM 3186) was purchased from Sigma-Aldrich, USA. This CRM was diluted 100 times with deionized water and employed for method validation.

### Instruments and apparatus

Fourier-transform infrared spectrometry (FT-IR) (Nicolet 6700 FT-IR, Thermo Scientific, USA), laser scattering particle size distribution analysis (Partica LA-950V2, Horiba, Japan), and transmission electron microscopy (TEM) (JEM-2010, Jeol, Japan) were employed for characterization of the synthesized Fe_3_O_4_@SiO_2_@C_18_ particles. Saturated magnetization (M_S_) and the hysteresis curve were analyzed using Vibrating Sample Magnetometer (VSM) (VSM 7404, Lakeshore Cryotonics, USA). X-Ray diffraction (XRD) patterns were obtained from JDX 3530, Jeol, Japan. The specific surface area (SSA) measurements were performed the Quantachrome® ASiQwin™ -Automated Gas Sorption Data analyser (Autosorb iQ, Quantachrome instrument, USA). The SSA values were calculated using the BET (Brunauer–Emmett–Teller) method. Orbital shaker (Unimax 2010, Heidolph, Germany) was used in the extraction step. A UV–Vis spectrophotometer (UV-1700, Shimadzu, Japan) and a 10-mm path length optical glass micro-cuvette (700 µL internal volume) (Hellma, Germany) were used for measurement of absorbance. Unbranded rectangular magnetic bar (3.7 cm × 6.3 cm × 1.2 cm) with a magnetic flux density of 3200 G was purchased from a local distributor in Bangkok, Thailand.

### Synthesis of Fe_3_O_4_@SiO_2_@C_18_

#### Synthesis of Fe_3_O_4_

The Fe_3_O_4_ core was prepared by chemical co-precipitation^18^. FeCl_2_∙4H_2_O 1.0 g and 2.7 g of FeCl_3_∙6H_2_O were dissolved together in 50 mL deionized water. Then the mixture was transferred into a 250-mL three-neck round bottom flask and heated to 80 °C under N_2_ atmosphere. To produce Fe_3_O_4_ black precipitate, 25 mL of 30% (w/v) NH_3_ solution were added into the mixture using a plastic syringe. After 20 min of reaction time, the black precipitate was washed several times with deionized water to remove excess NH_3_, followed by ethanol and then dried at 60 °C for 24 h.

#### Synthesis of Fe_3_O_4_@SiO_2_ and Fe_3_O_4_@SiO_2_@C_18_

First, 0.50 g of the Fe_3_O_4_ was sonicated in a mixture of ethanol and deionized water (80:20) for 10 min. Then under N_2_ atmosphere, 1 mL of 30% (w/v) NH_3_ solution and 1 mL of TEOS were added to the mixture under mechanical stirring. After 4 h of reaction time, the Fe_3_O_4_@SiO_2_ particles were washed several times with deionized water to remove excess reagents, followed by ethanol to remove the remaining water, and then dried at 60 °C for 24 h.

Chemical modification of the Fe_3_O_4_@SiO_2_ surface with C_18_ groups was carried out under N_2_ atmosphere by dispersing 0.50 g of Fe_3_O_4_@SiO_2_ in pyridine 40 mL^22^. Then, 1 mL of chloro(dimethyl)octadecylsilane was added to the mixture under continuous sonication for 20 min. The mixture was left at room temperature for 24 h to complete the reaction. The Fe3O4@SiO2@C18 particles were washed several times with ethanol before drying with N2.

### Adsorption capacity, reusability, and storage durability of the synthesized Fe_3_O_4_@SiO_2_@C_18_

The adsorption capacity (*q*_*e*_) of the synthesized Fe_3_O_4_@SiO_2_@C_18_ towards PMB–CTAB was studied. A PMB–CTAB solution containing 100 µg P L^−1^ was prepared by mixing 50 µL of the stock standard *o*-PO_4_^3−^ solution with 1 mL of each reagent (R_1_, R_2_, and CTAB) and made up with deionized water in a 50-mL volumetric flask^10^. The mixture was left for 15 min to ensure equilibrium PMB–CTAB formation. The adsorption capacity determination was carried out by dispersing 2 mg of Fe_3_O_4_@SiO_2_@C_18_ in 20 mL of the PMB–CTAB solution for 15 min. After magnetic decantation, the concentration of *o*-PO_4_^3−^ in the aqueous phase was quantified using external standard method. The adsorption capacity (*q*_*e*_, µg P g^−1^) of the synthesized Fe_3_O_4_@SiO_2_@C_18_ was estimated by using relationship *q*_*e*_ = (*C*_0_* − C*_*e*_)*V*/*m*, where *C*_*0*_ is the initial concentration of *o*-PO_4_^3−^ (µg P L^−1^), *C*_*e*_ is the concentration of *o-*PO_4_^3−^ after magnetic decantation (µg P L^−1^), *V* is the volume of the PMB–CTAB solution (L), and *m* is the mass of the Fe_3_O_4_@SiO_2_@C_18_ (g).

To investigate the reusability of the synthesized Fe_3_O_4_@SiO_2_@C_18_, the adsorption capacities of the Fe_3_O_4_@SiO_2_@C_18_ were compared after a number of uses. The Fe_3_O_4_@SiO_2_@C_18_ was washed with acidified ethanol, methanol, and deionized water between each usage cycle. Storage durability of the synthesized Fe_3_O_4_@SiO_2_@C_18_ was investigated by evaluating the adsorption capacity after storage over three months.

### Water sample preparations and d-MSPE procedure

Five freshwater samples were collected: three from river, one from canal, and one from tap water. The *o*-PO_4_^3−^ concentration of the samples was determined using the proposed d-MSPE method (Fig. 1). All samples were filtered through 0.45 µm syringe membrane filters (CN-CA, Chromex Scientific, UK) prior d-MSPE.

First, samples were pipetted into a 25-mL volumetric flask; 2.5 mL river water 1, 12.5 mL for river water 2 and 20 mL for the rest of samples, due to the difference analyte concentrations. Follow by 0.5 mL of each reagent (R_1_, R_2_, and CTAB) were sequentially added. Then, the mixture was left for 2.5 min at room temperature to form the PMB–CTAB ion pair complex before transferring 20 mL of the solution into a 150-mL Erlenmeyer flask which already contain 2 mg of Fe_3_O_4_@SiO_2_@C_18_. The mixture was shaken using an orbital shaker at 200 rpm for 5 min. The external magnet was used in order to isolate the PMB–CTAB-magnetic particles from the aqueous solution. The PMB–CTAB complex was then eluted from the particles by adding 500 µL of acidified ethanol and shaking for 1 min. After separation of Fe_3_O_4_@SiO_2_@C_18_ from eluate, the absorbance of PMB–CTAB complex in the acidified ethanol was measured using a UV–Vis spectrophotometer at 800 nm, which is the wavelength of maximum absorbance of the complex solution (Supplementary Materials Fig. S1).

## Results and discussion

### Characterization of the materials and adsorption capacity

**Figure 2:**
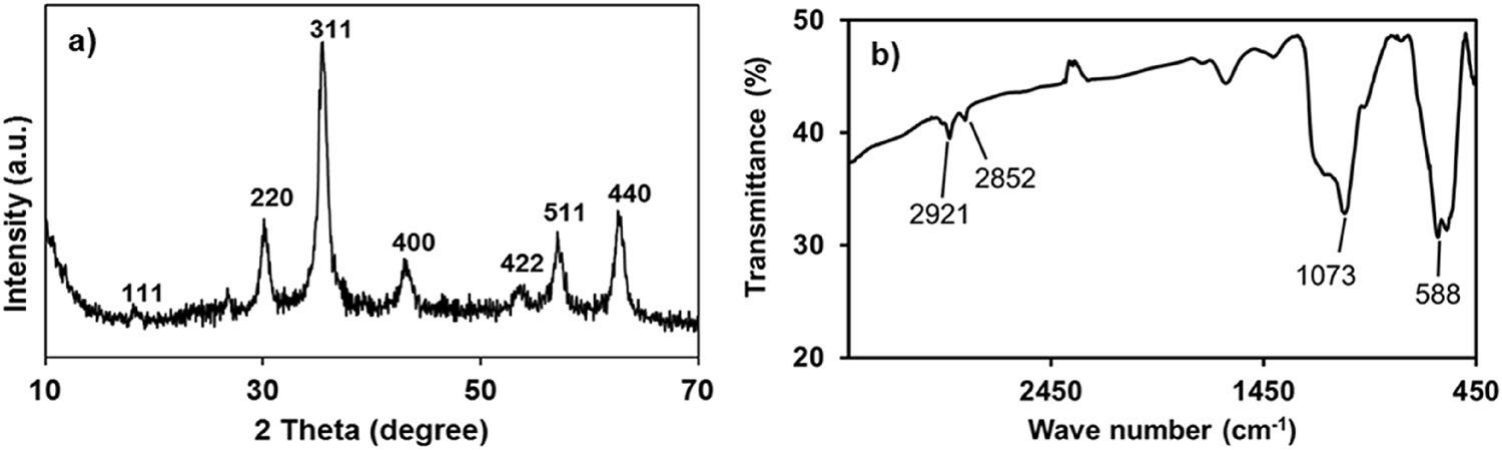
(**a**) XRD patterns of Fe_3_O_4_ particles and (**b**) FT-IR spectra of Fe_3_O_4_@SiO_2_@C_18_ particles at room temperature.

The crystal structure of the Fe_3_O_4_ material was analysed using XRD. The patterns are shown in Fig. 2(a) revealing five main peaks, which were indexed to the (1 1 1), (2 2 0), (3 1 1), (4 0 0), (4 2 2), (5 1 1), and (4 4 0), confirming the characteristic diffraction peaks of magnetite (Fe_3_O_4_) (ICDD: PDF 01–071-6336) with a face-centred cubic magnetite structure. Surface functionality of the Fe_3_O_4_@SiO_2_@C_18_ material was investigated using FT-IR (Fig. 2(b)). Absorption peaks at 588, 1073, 2852, and 2921 cm^−1^ representing characteristic vibrations of Fe–O–Fe, Si–O–Si, and C–H bonds of –CH_2_– and –CH_3_, respectively. This was in good agreement with the results reported in^27^, and confirmed that Fe_3_O_4_@SiO_2_@C_18_ had been successfully synthesized.

**Figure 3:**
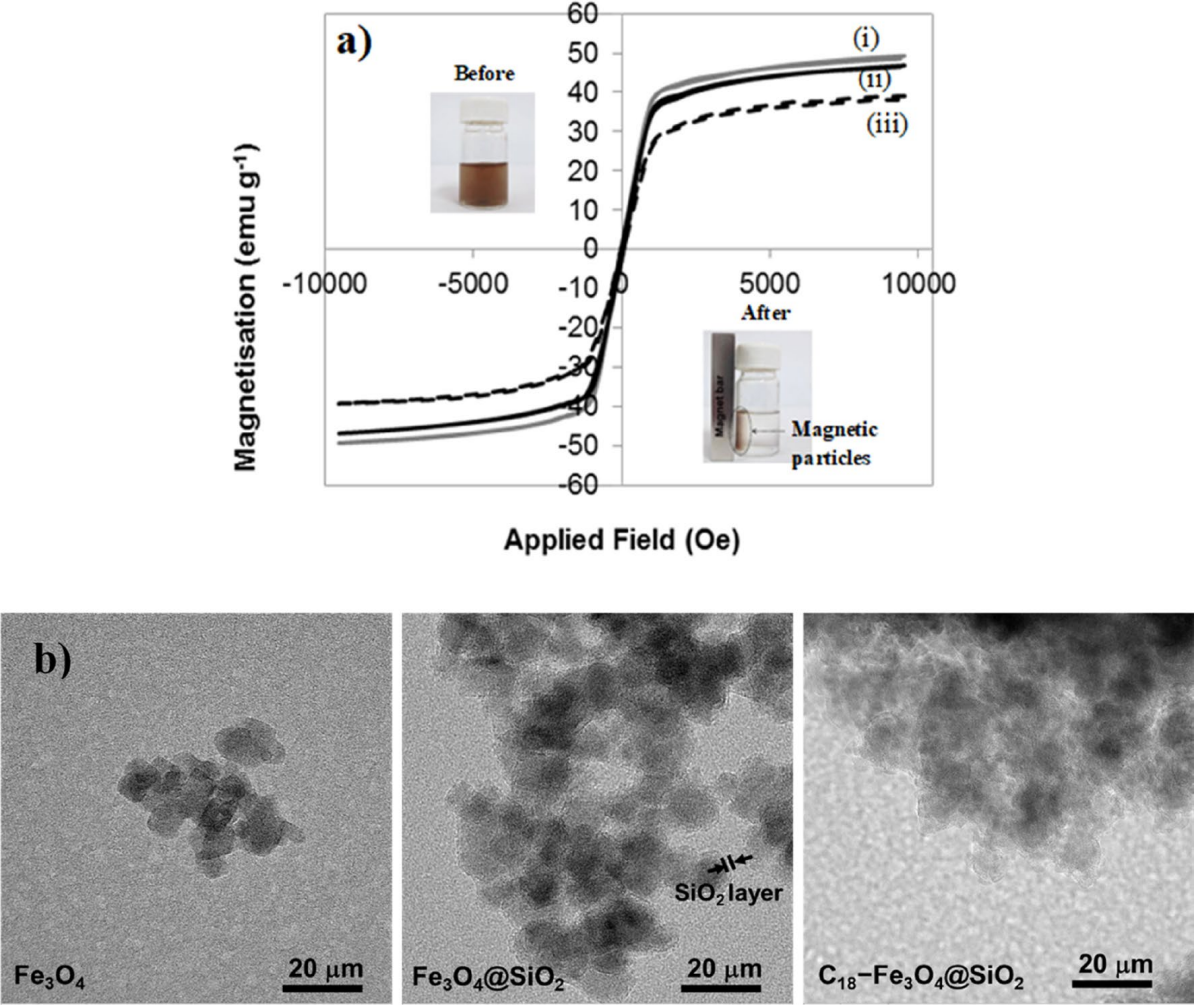
(**a**) Magnetic hysteresis curves of (i) Fe_3_O_4_, (ii) Fe_3_O_4_@SiO_2_, and (iii) Fe_3_O_4_@SiO_2_@C_18_ particles at room temperature. The images in the insets show Fe_3_O_4_@SiO_2_@C_18_ particles in an aqueous solution before and after separation using an external magnetic field. (**b**) TEM image of Fe_3_O_4_, Fe_3_O_4_@SiO_2_, and Fe_3_O_4_@SiO_2_@C_18_.

The magnetic properties, including magnetic hysteresis loops of the Fe_3_O_4_, Fe_3_O_4_@SiO_2_, and Fe_3_O_4_@SiO_2_@C_18_ particles, were determined using VSM at room temperature. Figure 3(a) shows the magnetization curves of all samples, with saturation magnetization (M_s_) values of 49.35 for Fe_3_O_4_, 46.88 for Fe_3_O_4_@SiO_2_ and 39.19 emu g^−1^ for Fe_3_O_4_@SiO_2_@C_18_. The magnetic saturation of the Fe_3_O_4_ slightly decreased after coating with silica and surface functionalization with C_18_ molecules. The hysteresis loops of all samples exhibited low coercive field and remanence values, indicating that the synthesized material had a superparamagnetic property at room temperature. The inset images in Fig. 3(a) show good dispersion of the Fe_3_O_4_@SiO_2_@C_18_ particles in aqueous solution and the separation of the Fe_3_O_4_@SiO_2_@C_18_ particles from the aqueous phase by the external magnet can simply done within a few minutes. The rapid magnetic response, simple separation from the aqueous phase, and re-dispersion of the Fe_3_O_4_@SiO_2_@C_18_ particles make it a suitable material for determining *o*-PO_4_^3−^ in water.

The morphology of the Fe_3_O_4_@SiO_2_@C_18_ particles compared with Fe_3_O_4_ and Fe_3_O_4_@SiO_2_ particles were next investigated using TEM (Fig. 3(b)). Morphology of Fe_3_O_4_ was found as slightly irregular shape with an average size of iron oxide about 7.5 ± 0.8 nm. After coating Fe_3_O_4_ by silica (SiO_2_) layer using TEOS and ammonium hydroxide, the size of Fe_3_O_4_@SiO_2_ particles was slightly increased with an average thickness of SiO_2_ layer approximately 2.0 ± 0.2 nm. After C_18_-functionalization to produce Fe_3_O_4_@SiO_2_@C_18_, agglomeration of the particles with irregular shape was observed. This could be caused by degradation of C_18_-molecules by using TEM (JEOL, JEM-2010) operated with high-voltage electron (200 kV)^28^. The average median and mean of particles sizes acquired by particle size distribution were 4.79 ± 3.35 µm and 10.12 ± 7.38 µm respectively. The particle size of Fe_3_O_4_@SiO_2_@C_18_ particles obtained from the particle size distribution analyser (Fig. S2) were larger than the particle size which obtained by TEM (Fig. 3(b)). This was cause by an agglomeration of hydrophobic Fe_3_O_4_@SiO_2_@C_18_ particles during size measurement in suspension form in water. The specific surface area of the Fe_3_O_4_@SiO_2_@C_18_ particle was also determined and found as 87.07 ± 19.49 m^2^ g^−1^, which was an average value obtained from five synthesized batches. The data of N_2_ gas adsorption isotherm of the particles were included in Supplemental information (Fig. S3). The obtained BET surface area value is relatively comparable with other reported values for mesoporous Fe_3_O_4_ systems^19,20^.

Reproducibility of the in-laboratory Fe_3_O_4_@SiO_2_@C_18_ was studied through the adsorption towards PMB–CTAB. The mean adsorption capacity (*q*_*e*_) of the Fe_3_O_4_@SiO_2_@C_18_ across six preparation batches was 314.3 ± 59.4 µg P g^−1^ (Table S1). The relative standard deviation (RSD) of the mean *q*_*e*_ showed acceptable HORRAT_r_^29^ precision of 1.7, which indicated that in-laboratory preparation of the Fe_3_O_4_@SiO_2_@C_18_ was reproducible.

### Influential parameters of dispersive magnetic solid-phase extraction (d-MSPE)

To develop a simple but yield good sensitivity protocol for determination of trace *o*-PO_4_^3−^ in freshwater, the key d-MSPE parameters were identified by optimizing one parameter at a time. Following the d-MSPE procedure shown in Fig. 1, 20 mL of standard solution containing 20 µg P L^−1^ were used to prepare a sample solution. When optimizing the sample volume, a standard solution of 13 nmol P was used.

#### Time and temperature for PMB–CTAB formation

**Figure 4:**
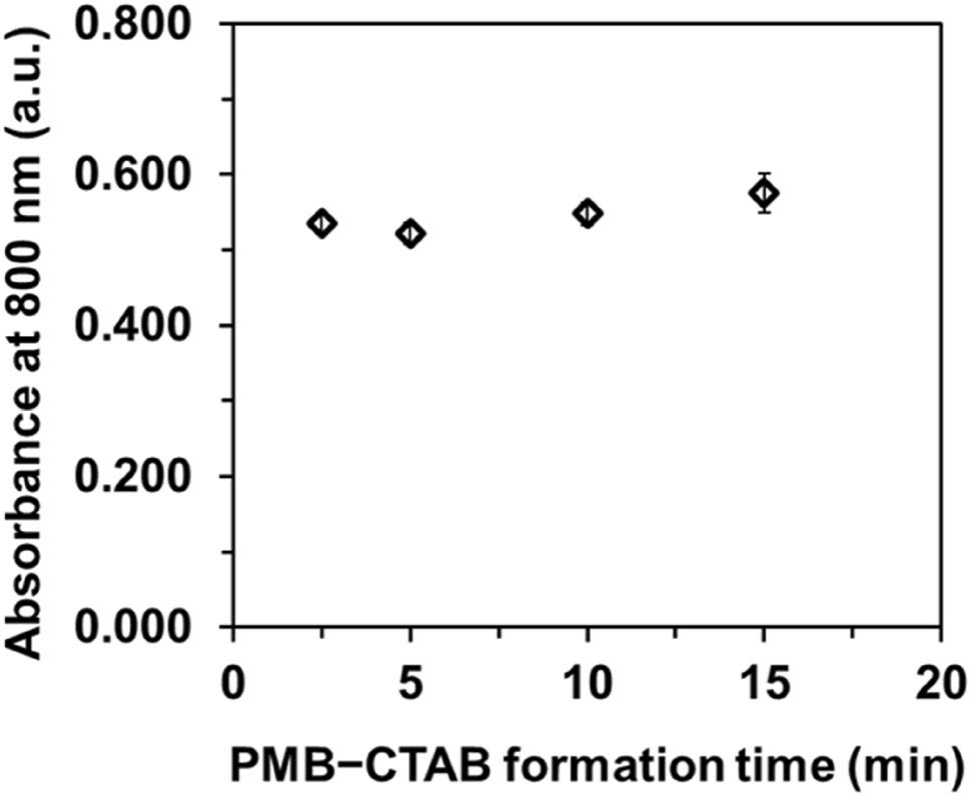
Investigation of PMB–CTAB formation time at room temperature before d-MSPE. Absorbance ± SD, *n* = 3.

The extraction efficiency of the Fe_3_O_4_@SiO_2_@C_18_ for PMB depended on the comprehensiveness coupled of PMB with CTAB. Since PMB is polyanion species, the mole ratio between phosphorus and CTAB was used at 1:18. Precipitation of PMB-CTAB were reported when using large amount of CTAB^30^ but in our study at 1:18 ratio, the PMB-CTAB ion pairs were in aqueous solution. The optimum time for PMB–CTAB formation at room temperature (25 °C) was investigated by observing the change in absorbance of the extracted PMB-CTAB. Figure 4 showed the relatively steadiness absorbance of the complex over reaction times of 2.5 to 15 min. The steadiness absorbance exhibit that the PMB–CTAB complexes were form completely within 2.5 min. The presence of sufficient ascorbic acid and the trace amount of *o*-PO_4_^3−^ are the main reason for the reaction quickness^26,31^.

The molybdenum blue method is temperature dependent. Even though formation of the PMB complex is accelerated as the temperature is increased^26,31^ however the PMB complex also can decompose under high temperature environment^26,32^. In this work the PMB–CTAB complex is the target specie for Fe_3_O_4_@SiO_2_@C_18_ not the PMB. So increasing temperature above room temperature even though speed-up the formation of PMB but will not improve the stability of PMB–CTAB ion paired complex formation.

Therefore, reaction time of 2.5 min and reaction at room temperature were chosen to minimize the analysis time and to simplify the analytical procedures.

#### Type and concentration of eluent

**Figure 5:**
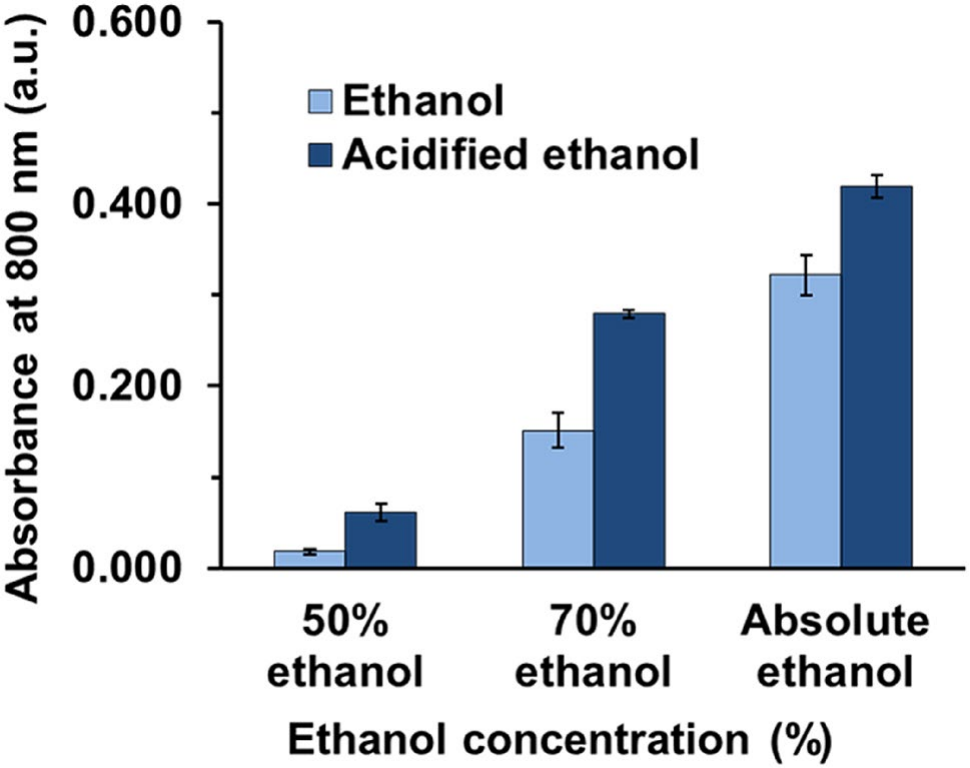
Investigation of PMB–CTAB extraction using different types and concentrations of eluent. Absorbance ± SD, *n* = 3.

Type and concentration of eluent should be optimized so it can effectively elute the PMB–CTAB complex from Fe_3_O_4_@SiO_2_@C_18_. Polar organic solvents have been shown to be effective for eluting^10,15^ the ion pair complex of phosphomolybdic acid and a cationic surfactant in an aqueous medium. Hence, ethanol was chosen for this work because of its compatible polarity, cost, availability and environment friendliness. Figure 5 shows the absorbance of the eluted PMB–CTAB using a mixed ethanol and water. As the concentration of ethanol was increased from 50% to ~ 99% (absolute ethanol), the absorbance, corresponding to the amount of eluted PMB–CTAB, increased. Furthermore, the addition of H_2_SO_4_ with a fixed final concentration of 0.56 mol L^−1^ to the ethanol solvent was found to enhance the eluting efficiency. This could be explained by the greater stability of the PMB–CTAB complex under acidic conditions (*i.e.* acidified ethanol). Polar solvent, H_2_SO_4_ and deionized water, cannot elute PMB–CTAB complex. Consequently, acidified ethanol was utilized in this work.

#### Sample solution volume and amount of Fe_3_O_4_@SiO_2_@C_18_ particles

**Figure 6:**
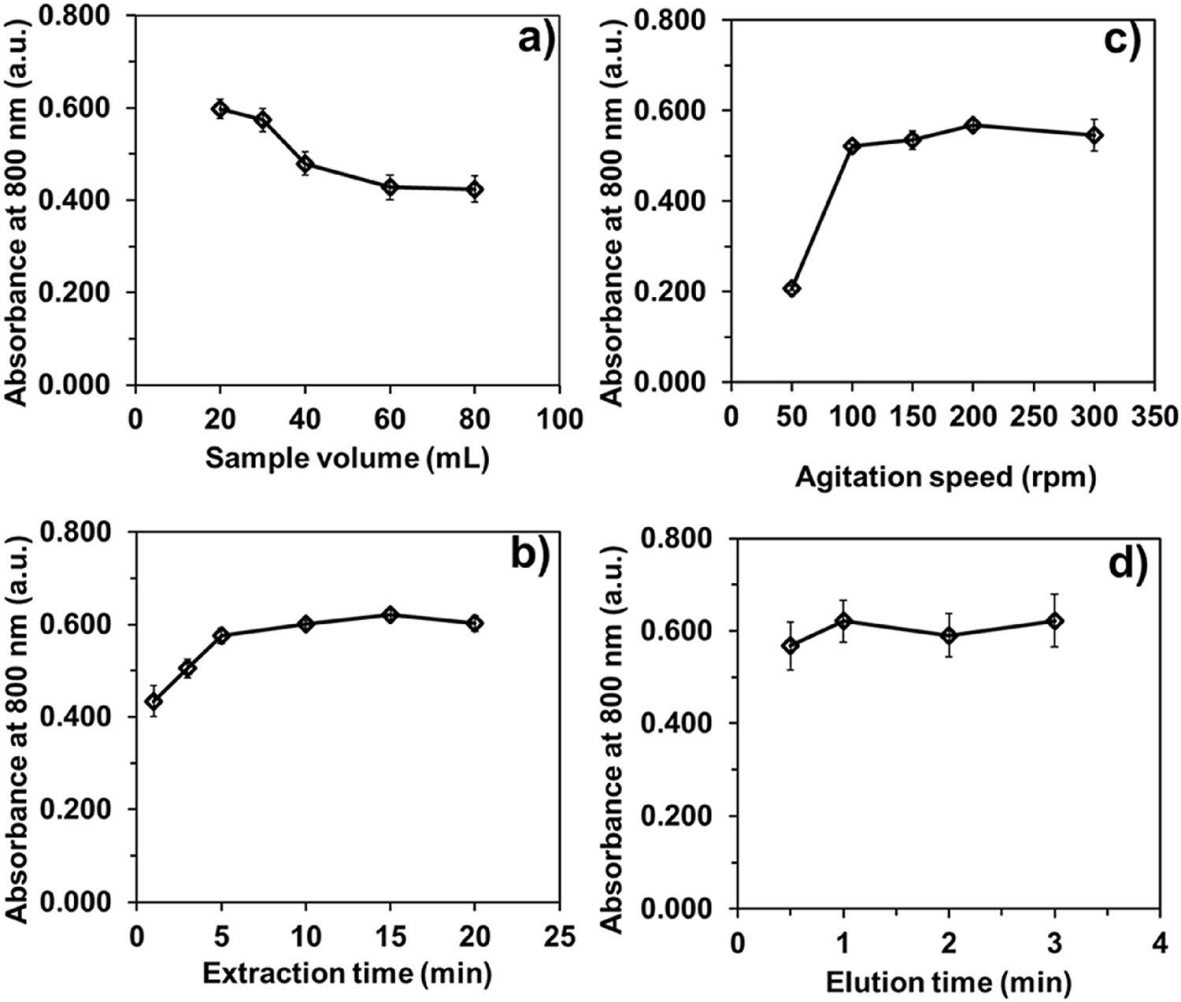
Effect of d-MSPE parameters; (**a**) sample volume, (**b**) extraction time, (**c**) agitation speed, and (**d**) elution time on absorbance signals. Absorbance ± SD, *n* = 3.

The effect of the solution volume on extraction efficiency was studied, since it determines the surface-to-volume ratio (contact area) between the solid sorbent and target analyte. Solutions with volumes ranging from 20 to 80 mL and containing a fixed amount of 13 nmol P, PMB–CTAB, were prepared. The quantity of Fe_3_O_4_@SiO_2_@C_18_ particles for each trial was 2 mg. Figure 6(a) shows that the absorbance values of the extracted PMB–CTAB changed significantly as the sample solution volume was increased. This reduction in absorbance was due to the decreasing in surface-to-volume ratio between the solution and the Fe_3_O_4_@SiO_2_@C_18_. A volume of 20 mL was therefore selected to provide a high surface-to-volume ratio.

Since the concentration of *o*-PO_4_^3−^ in clean natural water^1^ is lower than 50 µg P L^−1^. To investigate the minimum amount of Fe_3_O_4_@SiO_2_@C_18_ that adequate for detecting *o*-PO_4_^3−^ in clean natural water, 2 mg and 6 mg of Fe_3_O_4_@SiO_2_@C_18_ were dispersed in 20 mL of 30 µg P L^−1^ standard *o*-PO_4_^3−^ solution. Both 2 mg and 6 mg of Fe_3_O_4_@SiO_2_@C_18_ yielded not significantly difference in absorbance (Fig. S4). This result suggested that 2 mg of Fe_3_O_4_@SiO_2_@C_18_ was suffice for determination *o*-PO_4_^3−^ in clean natural water sample.

#### Extraction time and speed of agitation

Extraction of PMB–CTAB complexes from sample solution with Fe_3_O_4_@SiO_2_@C_18_ particles can be accelerated by agitation. Using of an ultra-sonication bath can create high frequencies agitation but also generates heat, which cause decomposition of PMB. The vortex mixer is a convenient method but suitable for low extraction volumes and yield small number of sample extraction throughput unless equip with special accessories. The orbital shaker produces less heat and more practical for a sample volume of 20 mL and was therefore used in this work. The extraction time and speed of shaking were studied in the following experiments.

At 200 rpm agitation speed and extraction times of 1 to 20 min were investigated. As results displayed in Fig. 6(b), the absorbance increased significantly from 1 to 5 min, then reached a plateau. This indicated that the PMB–CTAB was completely extracted by the Fe_3_O_4_@SiO_2_@C_18_ after 5 min of extraction. This extraction time was selected for investigating the speed of agitation.

The agitation speed determines the dispersion of Fe_3_O_4_@SiO_2_@C_18_ in the sample solution and can maximize the contact area within a given extraction time. In this work, the agitation speed was in the range of 50 to 300 rpm for 5 min. Figure 6(c) shows that the absorbance increased significantly as the speed of the shaker was increased from 50 to 100 rpm. Agitation speed over 100 rpm produced no further increase in absorbance, as the extraction of PMB–CTAB by Fe_3_O_4_@SiO_2_@C_18_ has already completed. An extraction speed of 200 rpm was therefore chosen to maintain the dispersion to maximize.

#### Elution time

To achieve a method with high sample throughput and yet high sensitivity, the optimum time for elution of the PMB–CTAB from the Fe_3_O_4_@SiO_2_@C_18_ was investigated. Aliquot of 500 µL acidified ethanol was used to elute the enriched PMB–CTAB. Figure 6(d) shows that the elution time of 1–3 min yielded no significantly different results. This indicated that the PMB–CTAB complex was eluted completely within 1 min. An elution time of 1 min was therefore selected.

### Interference studies

To determine the tolerance limit for common ions of the d-MSPE system in real sample matrices, different concentrations of possible interferences were spiked into a standard *o*-PO_4_^3−^ solution (10 µg P L^−1^). The absorbance values after addition of these species were compared with the control absorbance obtained from pure standard *o*-PO_4_^3−^. The tolerance limits in this work were defined as the change in ± 3SD of absorbance of the *o*-PO_4_^3−^ standard (10 µg P L^−1^). Table 1 shows the tolerance levels of various interference species using the proposed method, compared with other typical and recommended levels in different types of water.

**Table 1:** Typical and recommended level of the interference species in comparison with tolerance levels analysed by using the proposed method.

Species	Typical/recommended level	Type of water	References	Examined range	Tolerance level of this work
Silicate	0.368–3.68 mg Si L^−1^	River and lake water	^33^	4–6 mg Si L^−1^	6 mg Si L^−1^
	0.184 mg Si L^−1^	Sea water			
Arsenate	0.001–0.1 mg As L^−1^	Unpolluted water	^34^	0.001–0.01 mg As L^−1^	0.001 mg As L^−1^
Chromium (VI)	0.0005–0.002 mg Cr L^−1^	Surface water	^37^	0.05–0.5 mg Cr L^−1^	0.05 mg Cr L^−1^
	< 0.001 mg Cr L^−1^	Groundwater			
Nitrite	< 0.02 mg NO_2_^−^ L^−1^	Unpolluted water	^38^	10–70 mg NO_2_^−^ L^−1^	30 mg NO_2_^−^ L^−1^
Nitrate	< 1 mg NO_3_^−^ L^−1^	Surface water	^39^	10–100 mg NO_3_^−^ L^−1^	No interference in the studied range
Carbonate	25 to 400 mg CO_3_^2−^ L^−1^ as alkalinity	Ground and surface water	^40^	50–500 mg CO_3_^2−^ L^−1^	No interference in the studied range
Sulfate	0–230 mg SO_4_^2−^ L^−1^	Groundwater	^42^	250–1000 mg SO_4_^2−^ L^−1^	750 mg SO_4_^2−^ L^−1^
	2–250 mg SO_4_^2−^ L^−1^	Lake water			
	0–630 mg SO_4_^2−^ L^−1^	River water			
	2700 mg SO_4_^2−^ L^−1^	Sea water			
Sodium chloride	Up to 1000 mg NaCl L^−1^	Fresh water	^41^	1000–35,000 mg NaCl L^−1^	2300 mg NaCl L^−1^
	1000–3000 mg NaCl L^−1^	Fresh to brackish water			
	3000–5000 mg NaCl L^−1^	Brackish water			
	5000–35,000 mg NaCl L^−1^	Saline			
	35,000 mg NaCl L^−1^ and above	Hyper-saline			

Silicate (SiO_3_^2−^) and arsenate (AsO_4_^3−^) are two major interference ions of molybdenum blue method, because their chemical behavior and formation conditions are similar to those of *o*-PO_4_^3−^ ^2,26^. The results showed that the proposed d-MSPE tolerated the presence of SiO_3_^2−^ up to 6 mg Si L^−1^, which is higher than the level found in most natural waters^33^. However, in this study AsO_4_^3−^ was found to seriously hinder the analysis when presented even at the very low level of 0.001 mg As L^−1^ ^34^. Nonetheless, interference from the AsO_4_^3−^ can be eliminated by addition of thiosulphate (S_2_O_3_^2−^), reducing As (V) to As (III) which is not reactive with molybdenum blue^3,13,26^.

The oxidizing potential of hexavalent chromium (Cr (VI)) and nitrite ions (NO_2_^−^) allows them to interfere in the analysis of *o*-PO_4_^3−^. Since Cr (VI) can oxidize the ascorbic acid^35^ and NO_2_^−^ can oxidizes PMB to PMB product which has colorless solution^36^, false negatives might arise when these two species are present in the sample. It was determined that Cr (VI) above 0.05 mg Cr L^−1^ and NO_2_^−^ above 30 mg NO_2_^−^ L^−1^ caused significant reduction of absorbance. These levels of concentrations are found in polluted waters rather than in unpolluted waters^37,38^. Nitrate ions (NO_3_^−^) have been reported to be one of the possible interferences to the PMB reaction^2^. The proposed method can tolerate at least 100 mg NO_3_^−^ L^−1^, which is much higher than the typical level in most fresh waters^39^.

Interference from the other common species, carbonate (CO_3_^2−^)^40^, the dissolved sodium chloride (NaCl)^41^ and sulfate (SO_4_^2−^)^42^ had been studied. A level of 500 mg L^−1^ of CO_3_^2−^ did not altered the analytical results. Tolerance limits of interference from NaCl and SO_4_^2−^ salinities were found to be 2300 mg NaCl L^−1^ and 750 mg SO_4_^2−^ L^−1^, respectively. The tolerance levels of NaCl and SO_4_^2−^ interference in this study indicate our d-MSPE method allow the quantification of *o*-PO_4_^3−^ in freshwater but in high salinity waters such as brackish and seawaters, sample pretreatment *i.e*., dilution, precipitation with Ag^+^ will be needed before analysis.

Alkalinity of sample has been considered in the proposed d-MSPE method, since the PMB formation take place in highly acidic condition. Strong alkali sample might neutralize some of the acid in the reagent solution and effect the formation of PMB. Hence the acid amount in reagent solution had been compromised for the alkalinity of sample so the pH adjustment of sample could be neglect (pH range of unpolluted freshwater are between pH of 6–8).

### Reusability and storage durability of the Fe_3_O_4_@SiO_2_@C_18_ particles

The synthesized Fe_3_O_4_@SiO_2_@C_18_ could be reused at least three times without loss of PMB-CTAB adsorption capability (Fig S5(a)). After three cycles, the adsorption remarkedly decreased because of the hydrolysis of the silica bonded phase in the extremely acidic solutions (pH < 1) of the extraction and elution steps^43^.

The storage durability of the Fe_3_O_4_@SiO_2_@C_18_ particles was measured from the adsorption capacity of a representative batch. Fig S5(b) shows the adsorption in five cycles conducted over three months. These results clearly demonstrated the stability of the particles at least three months when stored in the desiccator at ambient temperature, as no reduction in adsorption capability was observed.

### Analytical performance

Under optimal conditions, the linear range of the proposed d-MSPE method was 1.0–30.0 µg P L^−1^ with a determination coefficient (r^2^) of 0.9925 (Fig. S6). The range covered the target *o*-PO_4_^3−^ levels in unpolluted water samples. The limit of detection (LOD) and limit of quantification (LOQ), calculated using 3 times and 10 times the standard deviation (SD) of the reagent blank divided by the slope of the calibration curve, were 0.3 and 1.0 µg P L^−1^, respectively. The attained LOD was satisfactory for detection of low levels of *o*-PO_4_^3−^ in unpolluted waters. The sensitivity enrichment was attained by calculating the slopes ratio of the calibration curve before and after d-MSPE (Absorbance = (0.0007 ± 0.0000)[P] + (0.0024 ± 0.0003); r^2^ 0.999 and Absorbance = (0.0224 ± 0.0007)[P] + (0.0976 ± 0.0148); r^2^ 0.998). A 32-fold enhancement was found after preconcentration using the proposed method. Reliability of the proposed method was evaluated by using the relative standard deviation (RSD) among six extraction batches, the results were 3.70% at 6.0 µg P L^−1^ and 2.49% at 25.0 µg P L^−1^. These %RSDs results indicated that the proposed method will yield sufficiently analytical precision.

We compared our method with the thirteen previously reported shown in Table 2. Comparison with d-MSPE method, LOD and linear range are comparable, but our method used shorter analysis time. Comparison when using bare Fe_3_O_4_ as sorbent even though much simpler than Fe_3_O_4_@SiO_2_@C_18_ but lack of acid tolerance has made the number of reusability become limited not to mention less reproducibility. Moreover, since bare Fe_3_O_4_ lack of specificity hence substantial amount of particles was needed and require extensive time for extraction. Compare with the nonmagnetic SPE methods and others, our method offers shorter analysis time than that of many methods. Relatively simpler than most methods, since our method doesn’t require heating or cooling during the blue complex formation or the extraction and preconcentration steps. The magnetic property of the Fe_3_O_4_@SiO_2_@C_18_ delivers fast collection of the solid phase not only with very simple procedures but also minimum loss of analytes.

**Table 2:** Comparison of analytical performance of the proposed d-MSPE method with other extraction/preconcentration methods for colorimetric determination of *o*-PO_4_^3−^ in water samples.

Method		Extractant phase	Sample solution volume (mL)	LOD (µg P L^−1^)	Enhancement factor	Linear range (µg P L^−1^)	Need of cooling or heating?	Analysis time (min)	References
**LLE**	CPE	Triton X-45	10	80.7	n.r	80.7–970.5	Yes	> 20	^3^
	CPE	Triton X-114	10	0.5	32.6	1–125	Yes	20	^4^
	SDME	MIBK	5	0.19	325	1.55–46.6	No	~ 7.5	^5^
	DLLME-SFODME	1-undecanol	25	0.23	50	1.24–18.6	Yes	18	^6^
	EISE	Layered-double hydroxides	10	5	14	5–200	No	18	^7^
	VA-NADES-ME	NADES	0.05	0.2	71	2–80	Yes^c^	13	^8^
	VA-SS-DLLME	NP4EO	0.06	0.1	50	0.5–28.0	No	10	^9^
**SPE (non-magnetic sorbent)**	d-SPE	TEPA-NCMs^a^	100	0.29	n.r	n.r	No	44	^14^
	On-line SPE-HPLC	ODS column^b^	50	0.05	n.r	0.05–33.0	Yes	30	^15^
	On-line SPE-FIA	C_18_–Sep-Pak^b^	195	0.048	n.r	0.099–1.50	Yes	30	^10^
	On-line SPE-SIA	HLB^b^	150	0.015	n.r	0.11–35.2	No	7–11	^11^
	On-line SPE-FA	HLB^b^	50	0.031	n.r	0–2.5	No	4–7	^12^
**SPE (magnetic sorbent)**	d-MSPE	Fe_3_O_4_^a^	5	0.3	40	0.77–32.3	No	23	^13^
	d-MSPE	Fe_3_O_4_@SiO_2_@C_18_^a^	20	0.3	32	1.0–30.0	No	8.5	This work

LLE: liquid–liquid extraction; CPE: cloud-point extraction; SDME: suspended droplet micro-extraction; DLLME-SFODME: dispersive liquid–liquid micro-extraction-solidified floating organic drop micro-extraction; EISE: electrostatically induced stoichiometric extraction; VA-NADES-ME: vortex-assisted natural deep eutectic solvent micro-extraction; VA-SS-DLLME: vortex-assisted based supramolecular solvents-dispersive liquid–liquid micro-extraction; SPE: solid-phase extraction; d-SPE: dispersive solid-phase extraction; TEPA-NCMs: Tetraethylenepentamine-functionalized nano-size meterials; FIA: Flow injection analysis; SIA: Sequential injection analysis; FA: flow analyzer; MIBK: Methyl isobutyl ketone; NADES: natural deep eutectic solvents; NP4EO: non-ionic nonylphenol tetra-ethoxylate ; HLB: hydrophilic-lipophilic balance; HPL: high performance liquid chromatography; ODS: octadecylsilane; LOD: limit of detection; n.r.: Not reported; ^a^: in-laboratory synthesized sorbent; ^b^: commercial sorbent; ^*c*^: requires heating to prepare NADES.

### Method validation and application to water samples

The developed d-MSPE method was validated by analysis of a certified reference material (CRM). The experimental mean value of 0.764 ± 0.0319 mg P L^−1^ (n = 3) was obtained for the CRM values of 0.752 ± 0.0140 mg P L^−1^. Statistical analysis *t*-test shows insignificantly difference between the certified value and the experimental mean value at 95% confidence level (*t*_stat_ 0.64 < *t*_crit_ 4.30). This confirmed accuracy of the developed d-MSPE method. The method was finally used to analyze *o*-PO_4_^3−^ in the five real samples (three river, one canal, and one tap water). Table 3 shows the *o*-PO_4_^3−^ concentrations in the water samples and recovery percentages analyzed using the proposed method. High recovery percentages ranging from 89.1 to 101.5% were obtained (Supplementary Materials Table S2), indicating that no matrices affected detection in real applications.

**Table 3:** Analyzed concentrations and recovery percentages of *o*-PO_4_^3−^ in water samples.

Sample (pH)	Analyzed *o*-PO_4_^3−^ ± SD, µg P L^−1^, (*n* = 3)	Recovery, %
River water 1 (pH 7.52)	60.5 ± 2.4	95.5 ± 21.5
River water 2 (pH 6.68)	12.0 ± 0.8	95.5 ± 14.6
River water 3 (pH 6.72)	2.3 ± 0.6	101.5 ± 15.6
Canal water (pH 8.53)	n.d	100.0 ± 1.8
Tap water (pH 7.38)	1.1 ± 0.1	89.1 ± 12.0

n.d.: not detectable (< LOD 0.3 µg P L^−1^).

## Conclusions

In this work, we developed a simple, fast, and highly sensitive method for trace analysis of *o*-PO_4_^3−^ in unpolluted freshwater by using silica coated magnetite functionalized with octadecyl (C_18_)silane (Fe_3_O_4_@SiO_2_@C_18_). The Fe_3_O_4_@SiO_2_@C_18_ was synthesized and characterized to confirm the final product. The synthesized particles were used for extraction and preconcentration of *o*-PO_4_^3−^ through hydrophobic interaction with PMB–CTAB ion pair complexes in the water. The proposed Fe_3_O_4_@SiO_2_@C_18_ d-MSPE method was operated at room temperature, using generic glassware and apparatus, short analysis time, simple extraction procedures, high sensitivity and detecting *o*-PO_4_^3−^ at concentrations as low as 0.3 µg P L^−1^, with a limit of quantification of 1.0 µg P L^−1^. The analysis was highly precise (RSD < 3.70%) and accurate (recovery 89.1–101.5%). Interference studies suggested the proposed method can tolerate a range of species commonly found in ordinary unpolluted freshwater samples. The Fe_3_O_4_@SiO_2_@C_18_ can store in desiccator at ambient temperature for over three months without impairing the adsorption capacity. These results emphasis the potential of our method for trace analysis of *o*-PO_4_^3−^ that could apply for various environmental samples.

## Supplementary Information

Supplementary Information.

## Abbreviations

CN-CA: Cellulose nitrate and cellulose acetate
CPE: Cloud-point extraction
CRM: Certified reference material
CTAB: Cetyl trimethyl ammonium bromide
DLLME-SFODME: Dispersive liquid–liquid micro-extraction with solidified floating organic drop micro-extraction
d-MSPE: Dispersive magnetic solid-phase extraction
d-SPE: Dispersive solid-phase extraction
DTAB: Dodecyltrimethylammonium bromide
EISE: Electrostatically induced stoichiometric extraction
FT-IR: Fourier-transform infrared spectrometry
HLB: Hydrophilic-lipophilic balance
HPLC: High performance liquid chromatography
LDHs: Layered-double hydroxides
LLE: Liquid–liquid extraction
LOD: Limit of detection
LOQ: Limit of quantification
MIBK: Methyl isobutyl ketone
NADES: Natural deep eutectic solvents
NP4EO: Non-ionic nonylphenol tetra-ethoxylate
n.r.: Not reported
ODS: Octadecylsilane
PMB: Phosphomolybdenum blue
PMB-CTAB: Phosphomolybdenum blue-cetyl trimethyl ammonium bromide
RSD: Relative standard deviation
SD: Standard deviation
SDME: Suspended droplet micro-extraction
SPE: Solid-phase extraction
SPE-FA: Solid-phase extraction-Flow analyzer
SPE-FIA: Solid-phase extraction-Flow injection analysis
SPE-HPLC: Solid-phase extraction-High performance liquid chromatography
SPE-SIA: Solid-phase extraction-Sequential injection analysis
TEM: Transmission electron microscopy
TEOS: Tetraethyl orthosilicate
TEPA-NCMs: Tetraethylenepentamine-functionalized nano-size composite materials
UV–Vis: Ultraviolet–Visible
VA-NADES-ME: Vortex-assisted natural deep eutectic solvent micro-extraction
VA-SS-DLLME: Vortex-assisted based supramolecular solvents-dispersive liquid–liquid micro-extraction
VSM: Vibrating Sample Magnetometer
XRD: X-Ray diffraction
