# Supplementary material for: Colorimetric determination of trace orthophosphate in water by using C18-functionalized silica coated magnetite
Source: Sci Rep. 2021 Nov 29;11:23073. doi: 10.1038/s41598-021-02516-4 (PMC8630040; doi:10.1038/s41598-021-02516-4)
Supplement: Supplementary file 1 — Supplementary Information. [file 41598_2021_2516_MOESM1_ESM.pdf]

Supplemental information

Colorimetric determination of trace orthophosphate in water by using C_{18}–functionalized silica coated magnetite

Vanpaseuth Phouthavong[1,2], Supone Manakasettharn[3], Duangkamon Viboonratanasri[3], Siriwit Buajarern[1], Panida Prompinit[3,*] and Kamonthip Sereenonchai[1,4,*]

[1]Department of Chemistry, Faculty of Science and Technology, Thammasat University, Pathumthani 12120, Thailand

[2]Department of Chemistry, Faculty of Natural Sciences, National University of Laos, P. O. Box 7322, Vientiane, Lao PDR

[3]National Nanotechnology Center, National Science and Technology Development Agency (NSTDA), Thailand Science Park, Pathumthani 12120, Thailand

[4]Flow Innovation-Research for Science and Technology Laboratories (FIRST Labs), Thailand

*Corresponding authors: panida@nanotec.or.th (P. Prompinit) and ksereenonchai@hotmail.com (K. Sereenonchai)

Supplementary Materials Figures (6) and Tables (2)

Fig. S1 Visible absorption spectra of PMB−CTAB in aqueous (solid line) and acidified ethanol (dash line) media.

Fig. S2 Particle size distribution of $Fe_3O_4@SiO_2@C_{18}$ sample. Samples obtained from four different batches of synthesis (Batch 01-04) were measured to calculate the average of particles size for both median and mean sizes and standard deviation (Std. Dev.) of particle size distribution. This study was carried out using Laser scattering particle size distribution analyzer LA-950, HORIBA.

Fig. S3 N_2 adsorption isotherm of five batches (01-05) of synthesis for $Fe_3O_4@SiO_2@C_{18}$ samples. The specific surface area (SSA) measurements were performed by the Quantachrome® ASiQwin™ -Automated Gas Sorption Data analyser. The SSA values were calculated using the BET (Brunauer–Emmett–Teller) method.

Fig. S4 The effect of amount of $Fe_3O_4@SiO_2@C_{18}$

Fig. S5 Investigation of a) Reusability of the synthesized $Fe_3O_4@SiO_2@C_{18}$ by considering adsorption capacity (q_e) of the magnetic particles after a number of uses and b) storage durability of the synthesized $Fe_3O_4@SiO_2@C_{18}$ in the desiccator at ambient temperature conditions.

Fig. S6 A calibration plots of the absorbance against *o*-PO_4^{3-} concentrations using the proposed d-MSPE method. Each point in the calibration was from triplicate measurements. The dash line represents reagent blank level.

Table S1. Adsorption capacity (q_e) of the $Fe_3O_4@SiO_2@C_{18}$ across six preparation batches.

Table S2. Recovery of *o*-PO_4^{3-} in water samples.

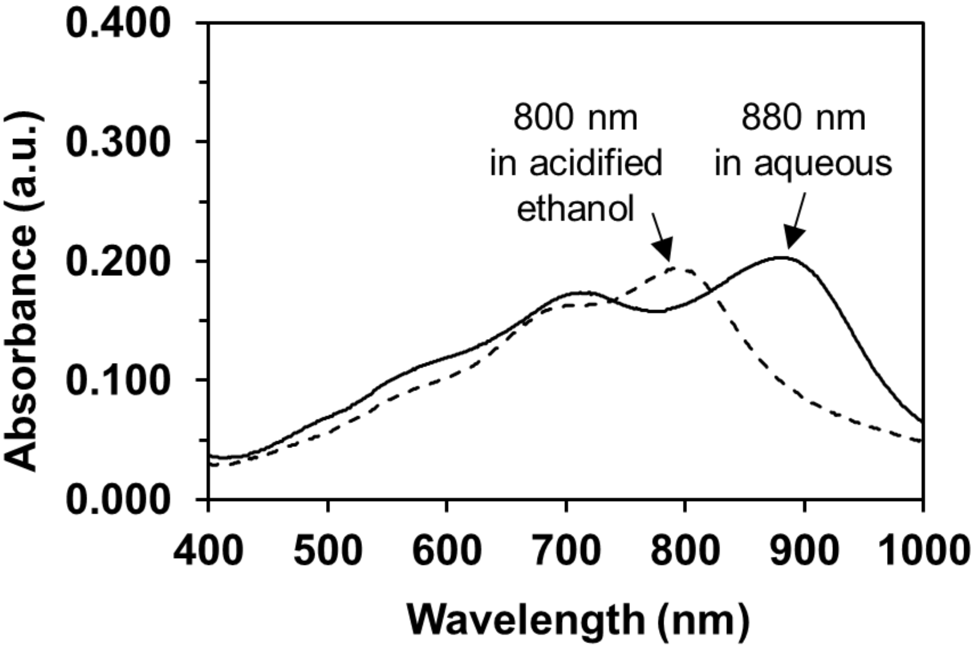

Fig. S1 Visible absorption spectra of PMB−CTAB in aqueous (solid line) and acidified ethanol (dash line) media.

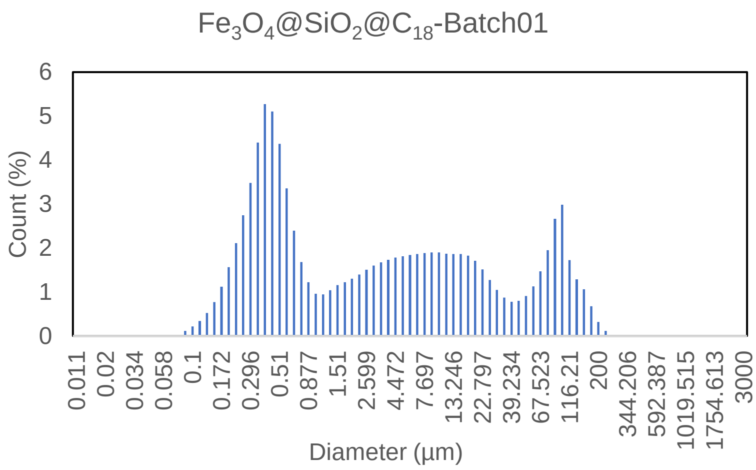

<u>Batch01</u>
Median size: 2.52 µm
Mean size: 19.81 µm
Std. Dev.: 36.27 µm

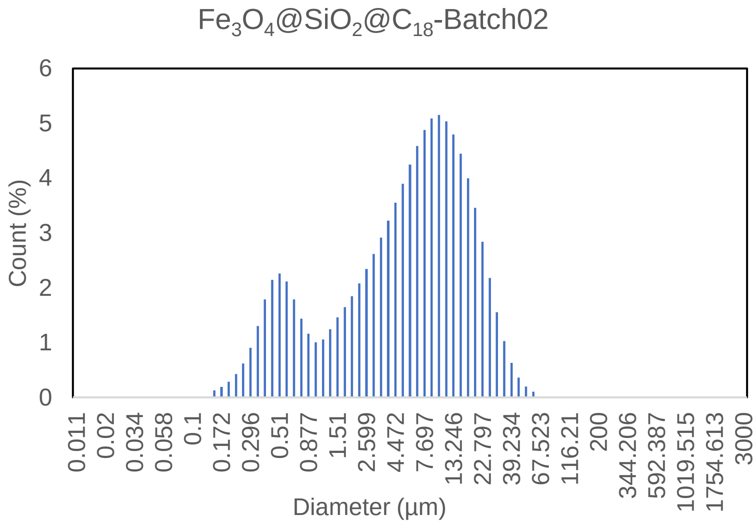

<u>Batch02</u>
Median size: 5.92 µm
Mean size: 8.18 µm
Std. Dev.: 8.08 µm

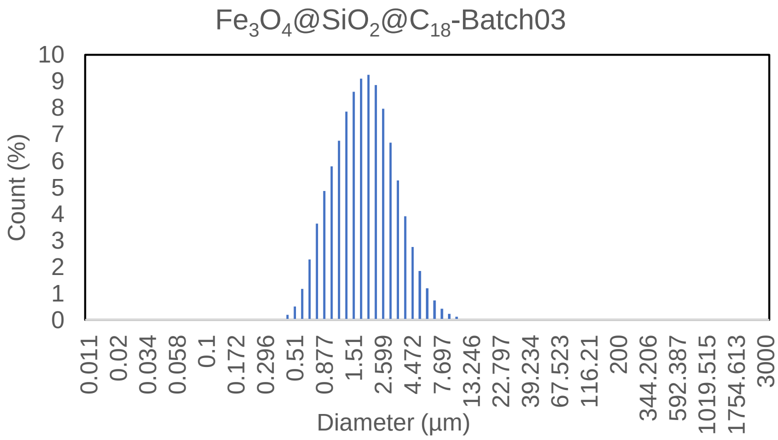

<u>Batch03</u>
Median size: 1.71 µm
Mean size: 2.01 µm
Std. Dev.: 1.22 µm

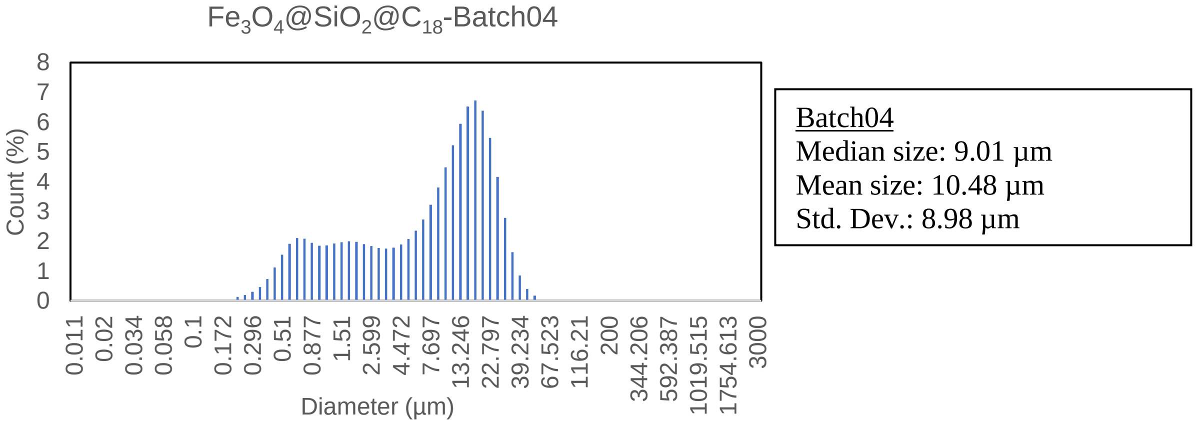

Fig. S2 Particle size distribution of $Fe_3O_4@SiO_2@C_{18}$ sample. Samples obtained from four different batches of synthesis (Batch 01-04) were measured to calculate the average of particles size for both median and mean sizes and standard deviation (Std. Dev.) of particle size distribution. This study was carried out using Laser scattering particle size distribution analyzer LA-950, HORIBA.

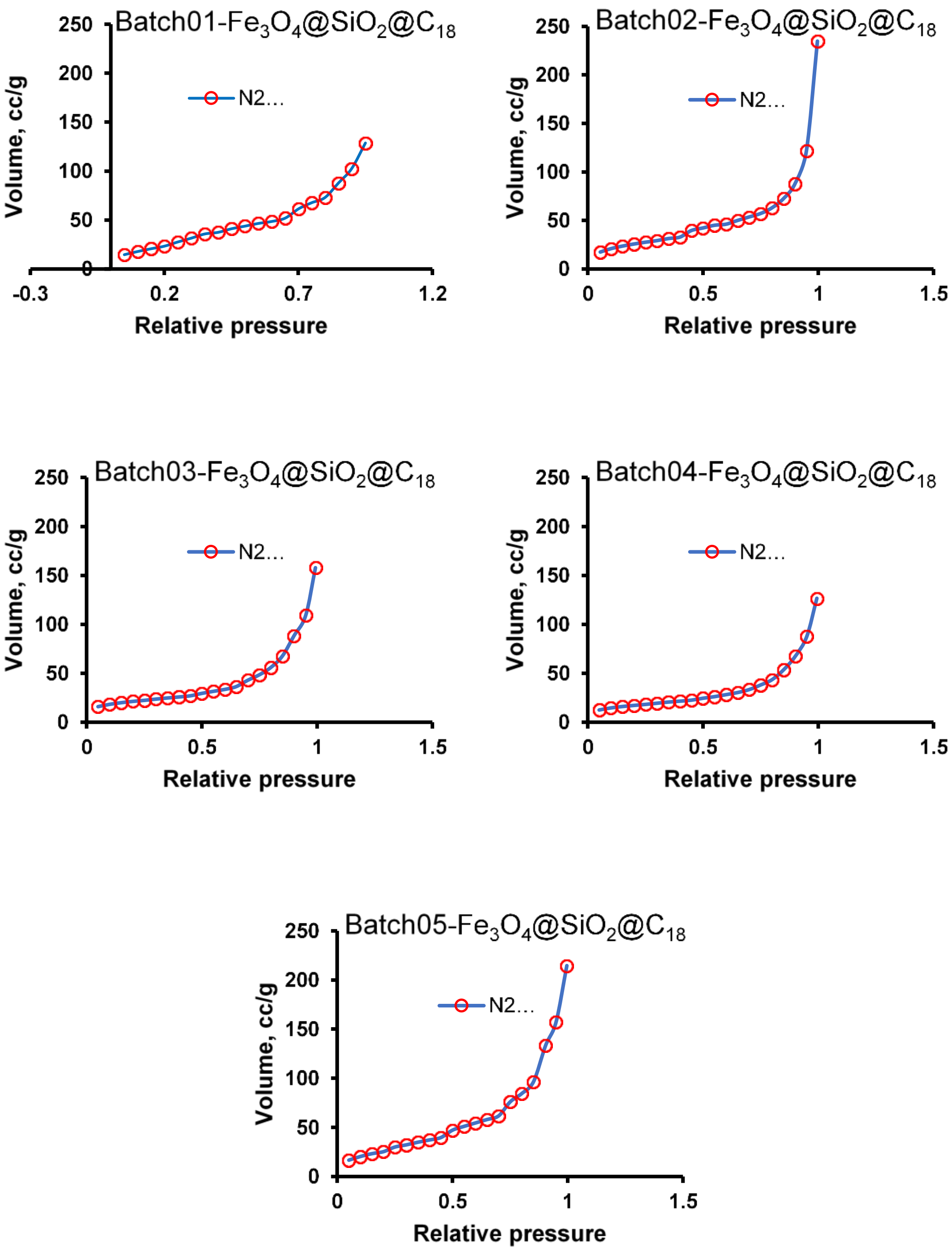

Fig. S3 N_2 adsorption isotherm of five batches (01-05) of synthesis for Fe_3O_4@SiO_2@C_{18} samples. The specific surface area (SSA) measurements were performed by the Quantachrome® ASiQwin™ -Automated Gas Sorption Data analyzer. The SSA values were calculated using the BET (Brunauer–Emmett–Teller) method.

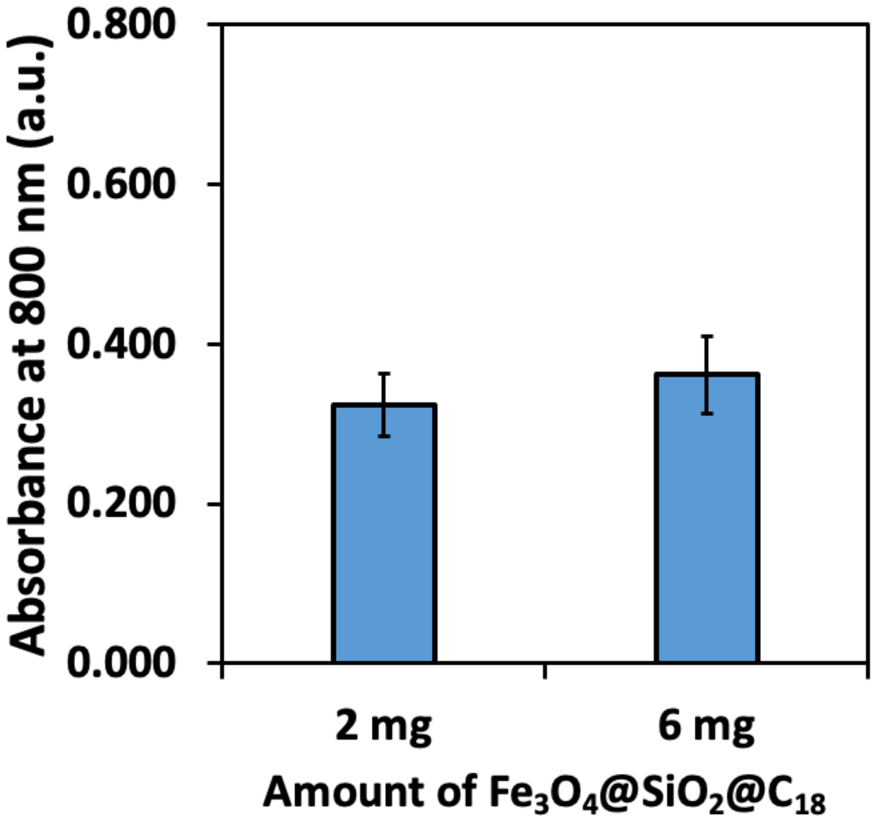

Fig. S4 The effect of amount of $Fe_3O_4@SiO_2@C_{18}$

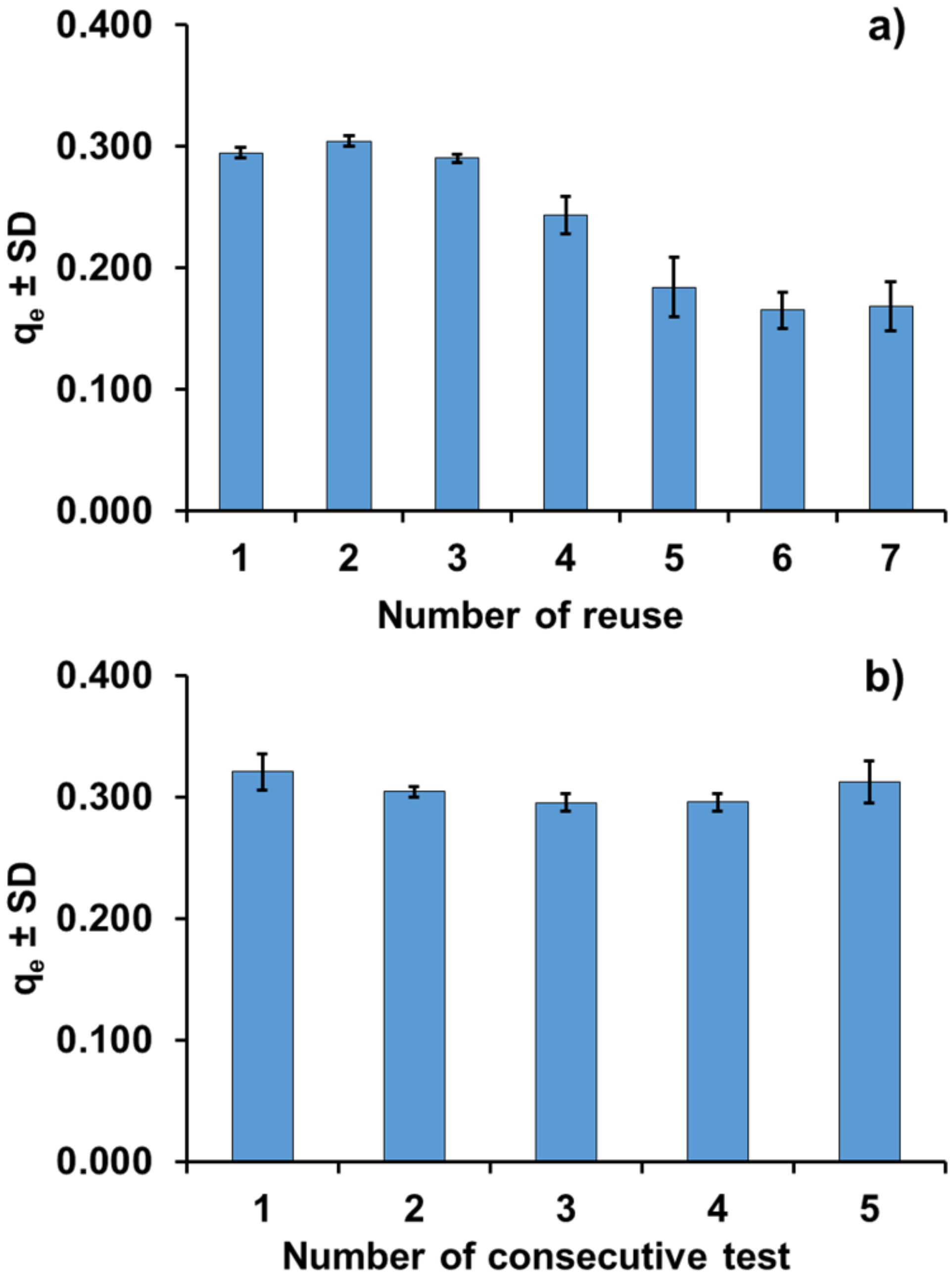

Fig. S5 Investigation of a) Reusability of the synthesized $Fe_3O_4@SiO_2@C_{18}$ by considering mean adsorption capacity (q_e, mg P g^{-1}) of the magnetic particles after a number of uses and b) storage durability of the synthesized $Fe_3O_4@SiO_2@C_{18}$ in the desiccator at ambient temperature conditions.

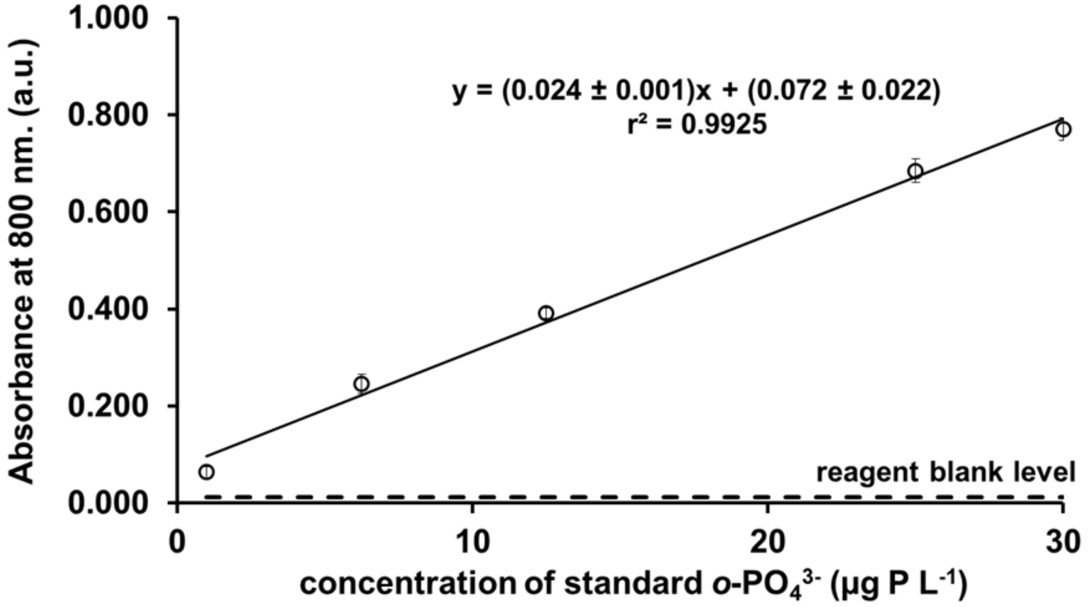

Fig. S6 A calibration plots of the absorbance against *o*-PO_4^{3-} concentrations using the proposed d-MSPE method. Each point in the calibration was from triplicate measurements. The dash line represents reagent blank level.

Table S1. Adsorption capacity (q_e) of the $Fe_3O_4@SiO_2@C_{18}$ across six preparation batches.

Number of synthesis batch	Mean q_e ± SD (µg P g^{-1})
1	298.2 ± 7.9
2	322.6 ± 10.5
3	261.9 ± 31.3
4	370.0 ± 43.0
5	321.0 ± 15.0
6	312.0 ± 17.5

Note: HorRAT(R) = 1.7, acceptable HorRat Values: 0.5 to 2.0 [29]

Table S2. Recovery of *o*-PO_4^{3-} in water samples.

Sample	Analyzed *o*-PO_4^{3-} ± SD, µg P L^{-1}, (*n* = 3)			
	Content	Added	Found	Recovery, %
River water 1	6.1 ± 0.2	2.9 ± 0.6	8.8 ± 0.2	95.5 ± 21.5
River water 2	5.6 ± 0.5	6.7 ± 0.2	12.0 ± 0.8	95.5 ± 14.6
River water 3	1.8 ± 0.4	6.7 ± 0.5	8.6 ± 0.8	101.5 ± 15.6
Canal water	n.d.	6.1 ± 0.1	6.2 ± 0.8	100.0 ± 1.8
Tap water	1.1 ± 0.1	6.4 ± 0.1	6.8 ± 0.1	89.1 ± 12.1

n.d. : not detectable (< LOD 0.3 µg P L^{-1})

Because the amount of *o*-PO_4^{3-} in water samples are significantly different, the quantity of each sample volume in the recovery study had to adjust accordingly. The river water 1 sample was 2.50 mL, 12.50 mL for river water 2 and aliquot of 20.00 mL for river water 3, canal water and tap water. The sample was mixed with R1, R2, CTAB (0.5 mL each), then adjusted to 25 mL with DI water before study.
